# A Simple Scoring System to Differentiate between Relapse and Re-Infection in Patients with Recurrent Melioidosis

**DOI:** 10.1371/journal.pntd.0000327

**Published:** 2008-10-29

**Authors:** Direk Limmathurotsakul, Wipada Chaowagul, Narisara Chantratita, Vanaporn Wuthiekanun, Mayurachat Biaklang, Sarinna Tumapa, Nicholas J. White, Nicholas P. J. Day, Sharon J. Peacock

**Affiliations:** 1 Mahidol-Oxford Tropical Medicine Research Unit, Faculty of Tropical Medicine, Mahidol University, Bangkok, Thailand; 2 Medical Department, Sappasithiprasong Hospital, Ubon Ratchathani, Thailand; 3 Center for Clinical Vaccinology and Tropical Medicine, Nuffield Department of Clinical Medicine, University of Oxford, Churchill Hospital, Oxford, United Kingdom; Charles Darwin University, Australia

## Abstract

**Background:**

Melioidosis is an important cause of morbidity and mortality in East Asia. Recurrent melioidosis occurs in around 10% of patients following treatment either because of relapse with the same strain or re-infection with a new strain of *Burkholderia pseudomallei*. Distinguishing between the two is important but requires bacterial genotyping. The aim of this study was to develop a simple scoring system to distinguish re-infection from relapse.

**Methods:**

In a prospective study of 2,804 consecutive adult patients with melioidosis presenting to Sappasithiprasong Hospital, NE Thailand, between1986 and 2005, there were 141 patients with recurrent melioidosis with paired strains available for genotyping. Of these, 92 patients had relapse and 49 patients had re-infection. Variables associated with relapse or re-infection were identified by multivariable logistic regression and used to develop a predictive model. Performance of the scoring system was quantified with respect to discrimination (area under receiver operating characteristic curves, AUC) and categorization (graphically). Bootstrap resampling was used to internally validate the predictors and adjust for over-optimism.

**Findings:**

Duration of oral antimicrobial treatment, interval between the primary episode and recurrence, season, and renal function at recurrence were independent predictors of relapse or re-infection. A score of <5 correctly identified relapse in 76 of 89 patients (85%), whereas a score ≥5 correctly identified re-infection in 36 of 52 patients (69%). The scoring index had good discriminative power, with a bootstrap bias-corrected AUC of 0.80 (95%CI: 0.73–0.87).

**Conclusions:**

A simple scoring index to predict the cause of recurrent melioidosis has been developed to provide important bedside information where rapid bacterial genotyping is unavailable.

## Author Summary

Melioidosis is a serious infectious disease caused by the Gram-negative bacterium, *Burkholderia pseudomallei*. This organism is present in the environment in areas where melioidosis is endemic (most notably East Asia and Northern Australia), and infection is acquired following bacterial inoculation or inhalation. Despite prolonged oral eradicative treatment, recurrent melioidosis occurs in approximately 10% of survivors of acute melioidosis. Recurrent melioidosis can be caused by relapse (failure of initial eradicative treatment) or re-infection with a new infection. The aim of this study was to develop a simple scoring system to distinguish between re-infection and relapse, since this has implications for antimicrobial treatment of the recurrent episode, but telling the two apart normally requires bacterial genotyping. A prospective study of melioidosis patients in NE Thailand conducted between 1986 and 2005 identified 141 patients with recurrent melioidosis. Of these, 92 patients had relapse and 49 patients had re-infection as confirmed by genotyping techniques. We found that relapse was associated with previous inadequate treatment and shorter time to clinical features of recurrence, while re-infection was associated with renal insufficiency and presentation during the rainy season. A simple scoring index to help distinguish between relapse and re-infection was developed to provide important bedside information where rapid bacterial genotyping is unavailable. Guidelines are provided on how this scoring system could be implemented.

## Introduction

Melioidosis, a serious Gram-negative infection caused by *Burkholderia pseudomallei*, is endemic across much of rural East and South Asia and in northern Australia [1]. The causative organism is present in the environment in these areas and infection is acquired by bacterial inoculation or inhalation. *B. pseudomallei* causes 20% of community-acquired septicemias in northeast Thailand [2], and is the most common cause of fatal community-acquired bacteremic pneumonia in Darwin, Australia [3]. Acute melioidosis is treated with parenteral treatment for at least 10 days, followed by oral treatment for 20 weeks [1]. The overall mortality of acute melioidosis is 50% in NE Thailand (35% in children), and 19% in Australia [1],[4]. Recurrent infection occurs despite 20 weeks of antimicrobial treatment and is the most important complication in survivors, affecting 13% of Thai patients who survive the primary episode [5]. A study that compared the bacterial genotype of strain pairs isolated during primary and recurrent melioidosis in over one hundred patients demonstrated that three quarters of cases were due to relapse (paired isolates had the same genotype), and one quarter were due to re-infection with a new strain [6]. Clinically this is an important distinction, with implications for epidemiology, investigation and management, but the overwhelming majority of medical centers treating patients with melioidosis in Asia do not have the facilities to perform bacterial genotyping and recurrence is usually considered to be synonymous with relapse. In addition, isolates from the primary episode are usually unavailable because bacterial strains are not routinely frozen. The purpose of this study was to define the association of readily accessible factors with relapse or re-infection, and to use these to develop a simple scoring system to help distinguish the most probable cause of recurrent melioidosis.

## Methods

### Patients

Study patients were adults (≥15 years) with culture-confirmed recurrent melioidosis who presented to Sappasithiprasong Hospital, Ubon Ratchathani, northeast Thailand between June 1986 and September 2005 and who were included in prospective studies of antimicrobial chemotherapy during this period. The standard of care throughout the study period was inpatient intravenous antimicrobial therapy, followed by a prolonged course of oral drugs. The prospective studies were either trials comparing parenteral antimicrobial regimens or trials comparing oral eradicative treatment regimens, as previously described (see [7] for list of published trials). Patients were followed up for recurrent melioidosis as a secondary outcome for trials comparing parenteral drugs and as a primary outcome for trials comparing oral treatment regimens.

Patients with suspected melioidosis were identified by twice-daily active case finding in the medical and intensive care wards. As part of eligibility screening for the clinical trials a history and examination was performed and samples taken for culture from suspected cases (blood culture, throat swab, respiratory secretions, pus or surface swab from wounds and skin lesions). Microbiology specimens were cultured for the presence of *B. pseudomallei*, as described previously [8]. Additional passive surveillance was undertaken via the diagnostic microbiology laboratory for patients on the surgical and pediatric wards with cultures positive for *B. pseudomallei*. All isolates were stored in trypticase soy broth with 15% glycerol at −80°C. A history and full clinical examination was performed on all cases of culture proven melioidosis. Details of history, examination, laboratory results, antimicrobial treatment and clinical course were maintained on a password protected computer database. Patients who survived the primary episode received oral eradicative treatment and were followed up monthly for one year, then yearly thereafter. Oral antimicrobial regimens were as described elsewhere [7]. Patients with recurrence were identified from the history, patient notes and by cross-reference with our database. Follow up data in this study was to February 2007. Ethical permission for all clinical trials was obtained from the Ethical and Scientific Review Subcommittee of Thai Ministry of Public Health. Patients gave written informed consent to participate in the trials.

### Genotyping and definition of relapse and re-infection

Single isolates obtained from the first and recurrent episode were compared using a combination of PFGE and MLST, as described previously [6],[9]. Recurrent melioidosis was defined as the development of new symptoms and signs of infection in association with a culture positive for *B. pseudomallei* following initial response to oral antibiotic therapy. Relapse and re-infection were defined on the basis of typing of isolates from the first and subsequent episode(s). Isolates from the same patient with an identical banding pattern on PFGE were considered to represent a single strain and these patients were classified as having relapse. Isolates from the same patient that differed by one or more bands were examined using a screening approach based on MLST, as described previously [6]. Isolates from the same patient with a different sequence type (ST) were classified as representing re-infection, while those with an identical ST were classified as representing relapse.

### Antimicrobial susceptibility testing

All *B. pseudomallei* isolates were tested for susceptibility to the antimicrobial drugs used to treat melioidosis (meropenem, ceftazidime, amoxicillin-clavulanic acid, chloramphenicol, doxycycline and trimethoprim/sulfamethoxazole (TMP-SMX)). This was performed using the disk diffusion method with the exception of TMP-SMX, which was assessed using the Etest (AB Biodisk, Solna, Sweden) [10]. All isolates defined as intermediate or resistant to a given drug by disk diffusion were tested further using the E-test. Interpretative standards were based on CLSI guidelines, which lists resistance for ceftazidime, amoxicillin-clavulanic acid, doxycycline and TMP-SMX as ≥32 mg/L, ≥32 mg/L, ≥16 mg/l and ≥4/76 mg/L, respectively, and intermediate resistance as 16 mg/L, 16 mg/L, 8 mg/l and N/A, respectively [11].

### Definitions

Diabetes mellitus was defined as either pre-existing, or a new diagnosis as defined by the American Diabetes Association criteria [12]. Impaired renal function was defined as an estimated glomerular filtration rate (GFR) below 60 mL/min/1.73 m^2^ at admission. GFR was estimated using an abbreviated form of the Modification of Diet in Renal Disease study equation [13]. Hypotension was defined as a systolic blood pressure less than 90 mmHg, acute renal failure as a 50% decrease in the baseline-calculated GFR [14], and respiratory failure as the need for mechanical ventilation. The time between the primary episode and recurrent episode was measured from the start of oral antimicrobial therapy to the clinical onset of culture-confirmed recurrent infection.

### Statistical analysis

The primary outcome of interest was cause of recurrent infection. Comparison between relapse and re-infection for each variable was performed using Fisher's exact test or the Wilcoxon-Mann-Whitney test, as appropriate. We selected potential predictor variables to study based on our collective clinical experience and information from other studies [5]–[7]. The variables considered included sex, age, diabetes, estimated GFR during recurrent infection, body sites involved in the primary and recurrent episode, complications of recurrent infection, antimicrobial treatment given for the primary episode, patterns of antimicrobial resistance for the primary and recurrent isolates, calendar month of presentation of recurrent episode, and duration between primary and recurrent episode. The creatinine level on recurrent episode was missing for 17 patients (12%) and the most recent creatinine levels during follow-up before the recurrent episode were used instead.

Variables associated with relapse/re-infection at p<0.20 were included as independent variables in a multivariable logistic regression model with relapse/re-infection as the dependent variable. Variables were removed one at a time from the model if the p-value as determined by the likelihood ratio test was >0.05, least significant variable first. To double check that no significantly predictive variables were removed during this process, each de-selected variable was tested in turn with the final model and reintroduced into the model if p<0.05 [15].

Variables in the final model were used to construct a scoring system. For simplicity, estimated GFR was categorized into four levels (<30, 30 to <60, 60 to <90 or ≥90) based on clinical practice guidelines [13]. Time to recurrent melioidosis was dichotomized (<1 year or ≥1 year) and duration of oral treatment received on primary episode was categorized into four levels (<8 weeks, 8 to <16 weeks, 16 to 20 weeks or >20 weeks) based on previous knowledge [6],[7]. These dummy variables were used in a multivariable logistic regression analysis. The coefficient for each variable was multiplied by 10 and rounded off to the nearest integer. A total score was calculated by summing the points from each variable for each patient, and the results plotted on a receiver-operator characteristic curve. The Hosmer-Lemeshow goodness-of-fit test was used to evaluate the regression model. Discrimination referred to the ability to distinguish re-infection from relapse, and was quantified by the area under receiver operating characteristic curves (AUC).

Bootstrap resampling procedures were used to assess the internal validity of the model and to adjust for over-fitting or over-optimism. The apparent performance of the scoring system (AUC) on the original data set may be better than the performance in another data set. One thousand random bootstrap samples were drawn with replacement from the original data set. The logistic regression model and scoring system generated from the bootstrap sample was evaluated in the bootstrap sample and in the original sample. The bootstrap sample set represented training data and the original sample set represented test data. The difference between the performances in both sets was an estimate of the optimism in the apparent performance. This difference was averaged to obtain a stable estimate of the optimism. The optimism was subtracted from the apparent performance to estimate the internally validated performance. All analyses were performed using the statistical software STATA/SE version 9.0 (StataCorp LP, College Station, Tx.).

## Results

A total of 2,804 adult patients with culture-confirmed melioidosis were seen during the 19-year study period. Of these, 1,401 (50%) adult patients died during admission. Of the adults who survived, 1,001 (71%) patients presented to follow up clinic at least once. Median duration of follow-up for patients without recurrence was 65 weeks (25^th^ percentile-75^th^ percentile, 22–179 weeks; range, 1–954 weeks). A total of 194 episodes of culture-confirmed recurrent melioidosis occurred in 170 (17%) patients. Of these, 148 (76%) strain pairs from the primary and recurrent episode were available for genotyping from 141 patients. Bacterial genotyping had been performed previously for 122 episodes in 115 patients [6], and genotyping of the remainder was performed during this study.

Of the 148 episodes of recurrent melioidosis, 98 episodes in 92 (65%) patients were defined by genotyping as relapse. Four of these patients relapsed twice and 1 patient relapsed three times. The other 50 episodes in 49 (35%) patients were due to re-infection. One patient had re-infection after completing treatment for an episode of relapse. For the purposes of this study, only the 141 first episodes of recurrent melioidosis (92 relapse and 49 re-infection) were analyzed.

All *B. pseudomallei* isolates associated with the primary episode of recurrent infection were susceptible to ceftazidime, amoxicillin-clavulanic acid and doxycycline, while 21/141 (15%) were resistant to TMP-SMX. All isolates associated with recurrence were susceptible to ceftazidime. Strains associated with re-infection were resistant to amoxicillin-clavulanic acid, doxycycline and TMP-SMX in 2% (1/49), 2% (1/49) and 16% (8/49) of cases, respectively, while, strains associated with relapse were resistant in 1% (1/92), 1% (1/92) and 12% (11/92), respectively (p>0.05, all). Two patients with relapse associated with the development of bacterial resistance to amoxicillin-clavulanic acid (MIC from 2 to 16 mg/L) or doxycycline (MIC from 1 to 96 mg/L) received antimicrobial treatment with the respective agent for at least 8 weeks prior to relapse.

**Figure 1:**
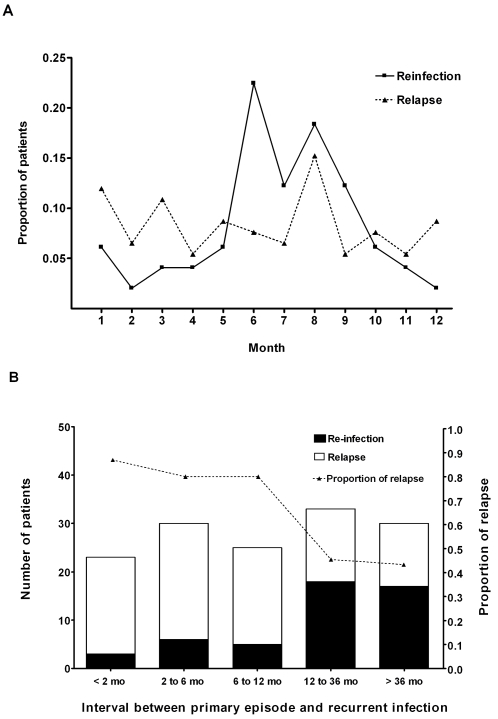
Comparison between patients with relapse and re-infection in relation to: (A) calendar month of presentation and (B) interval between primary episode and recurrent infection. Dotted line in Figure 1B shows the proportion of patients with relapse presenting within each interval (right Y axis).

The majority of patients with re-infection presented in the rainy season, the period of greatest melioidosis incidence, while patients with relapse presented throughout the calendar year without evident seasonality (p = 0.002, Figure 1A). Demographic characteristics and clinical features are shown in Table 1. Sex and age were comparable between the two groups. Diabetes mellitus was the most common underlying condition in both relapse and re-infection. Impaired renal function was present in 55 (60%) of 92 patients with relapse and 39 of 49 (80%) patients with re-infection (p = 0.02). Distribution of infection and organ involvement during primary infection and at time of recurrence was not different between patients with relapse and re-infection. There was no difference in severity of infection between relapse and re-infection as defined by hypotension, acute renal failure or respiratory failure (p>0.05 in all cases). Death occurred in 17 (18%) patients with relapse and 13 (27%) patients with re-infection (p = 0.29).

**Table 1:** Demographic characteristics.

Variable	Relapse (n = 92)	Re-infection (n = 49)	*P* value
Men, No. (%)	59 (64%)	29 (59%)	0.59
Age (yr) at recurrence, median (Q1–Q3)	49 (42–58)	47 (39–55)	0.25
Diabetes mellitus	58 (63%)	27 (55%)	0.37
Estimated GFR* on admission with recurrence, median (Q1–Q3)	53 (29–81)	40 (20–59)	0.02
Site(s) involved during recurrent infection			
Bacteremia	43 (47%)	28 (57%)	0.29
Pneumonia	27 (29%)	17 (35%)	0.57
Liver abscess	17 (18%)	9 (18%)	>0.99
Splenic abscess	14 (15%)	7 (14%)	>0.99
Skin or soft tissue infection	31 (34%)	16 (33%)	>0.99
Arthritis	13 (14%)	8 (16%)	0.81
Osteomyelitis	7 (8%)	1 (2%)	0.26
Complications of recurrent infection			
Hypotension	15 (16%)	11 (22%)	0.37
Acute renal failure	22 (24%)	17 (35%)	0.24
Respiratory failure	10 (11%)	7 (14%)	0.59
First oral antibiotic regimen for primary episode			
Three-drug regimen†	9 (10%)	6 (12%)	
Four-drug regimen‡	10 (11%)	12 (24%)	0.14
Amoxycillin-clavulanic acid	23 (25%)	12 (24%)	
Other regimen§	50 (54%)	19 (39%)	
Duration of oral treatment for primary episode, weeks, median (Q1–Q3)	1 (0–5)	16 (0–21)	<0.01
Recurrence in rainy season (June to November)	44 (48%)	37 (76%)	<0.01
Time to recurrence (months) median (Q1–Q3)	6 (2–17)	24 (9–45)	<0.01
Death attributable to recurrent melioidosis	17 (18%)	13 (27%)	0.29

Abbreviations: GFR, glomerular filtration rate; Q1–Q3, 25^th^ percentile and 75^th^ percentile; ^*^ mL/min per 1.73 m^2^, ^†^ Trimethoprim-sulfamethoxazole and doxycycline, ^‡^ Trimethoprim-sulfamethoxazole, doxycycline, and chloramphenicol, ^§^ Fluoroquinolone-based regimen, doxycycline alone, and trimethoprim-sulfamethoxazole alone.

On univariable analysis, the duration of oral antibiotic treatment for the primary episode was significantly shorter for patients with relapse than re-infection (p<0.001). The median time to relapse was also significantly shorter than time to re-infection (6 months versus 24 months, p<0.001) (Figure 1B). On multivariable analysis, significant independent predictors of re-infection were the presence of a low GFR on admission for the recurrent episode, an interval between the primary infection, and recurrence of more than one year and calendar period of presentation (rainy season). Short duration of oral antimicrobial treatment for first episode of infection was predictive for relapse (Table 2). The AUC for this model was 0.81 (95% CI: 0.74–0.89), and the Hosmer-Lemeshow goodness-of-fit test was not significant for lack of fit (Hosmer-Lemeshow statistics = 9.24, *df* = 8, p = 0.32,).

**Table 2:** Multivariable predictors of re-infection among patients with recurrent melioidosis.

Predictor	OR ‡ (95% CI)	*P* Value
Time to recurrent melioidosis more than one year	3.33 (1.44–7.70)	<0.01
Presentation in rainy season (June to November)	3.11 (1.30–7.47)	0.01
Duration of oral treatment received*	1.04 (1.01–1.08)	0.01
Estimated GFR on admission with recurrence (mL/min per 1.73 m^2^)†	0.83 (0.72–0.97)	0.02

Abbreviations: CI, confidence interval; OR, odds ratio.

*: The OR is for a week increase of treatment with effective oral treatment regimens, including TMP-SMX and doxycycline based regimens and amoxicillin-clavulanic acid regimen.

**†:** The OR is for a 10 mL/min per 1.73 m^2^ increase.

**‡:** Model Chi-square = 42.10; *df* = 4; *P*<0.001; area under ROC curve = 0.81 (95% CI: 0.74–0.89); Hosmer-Lemeshow statistics = 9.24, *df* = 8, *P* = 0.32.

**Figure 2:**
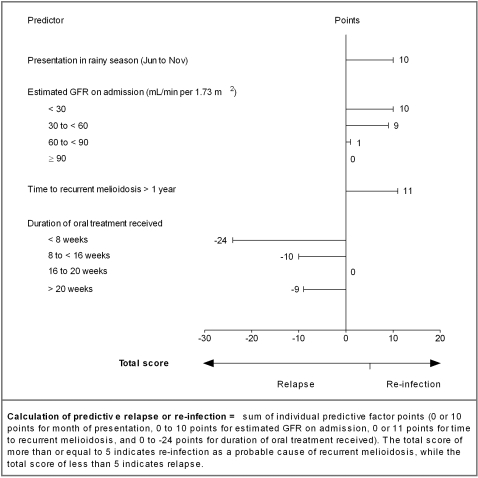
Four predictors of re-infection and relapse for patients with recurrent melioidosis. Points can be determined for each of the predictors using the figure. Factors associated with re-infection give a positive score, while factors associated with relapse give a negative score. The total score is reached by adding the points together for these four variables. A total score of more than or equal to 5 is predictive for re-infection as the probable cause of recurrent melioidosis, while a total score of less than 5 is predictive for relapse.

A scoring system was generated based on a combination of predictors of re-infection or relapse in the final logistic regression model (Figure 2). Factors associated with re-infection (time to recurrence more than one year, presentation during the rainy season or with reduced renal function) were given a positive score. Factors associated with relapse were given a negative score. A non-linear association was found between the duration of oral treatment received and predictive value of relapse. A score was reached based on the accumulation of points from the four variables. The AUC for the re-infection score was 0.80 (95%CI: 0.73–0.87) after applying the bootstrap correction.

**Figure 3:**
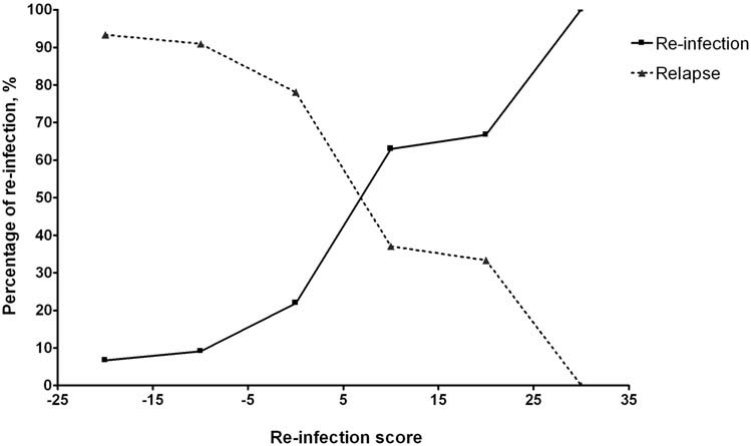
Predictive ability of the risk index model for relapse and re-infection of a total score within the range <−15, −15 to <−5, −5 to <5, 5 to <15, 15 to <25, and ≥25, respectively.

The predictive ability of the risk index model for relapse and re-infection is depicted in Figure 3. A score of less than 5 correctly identified relapse in 76 of 89 patients (85%) in this group, whereas a score of more than or equal to 5 correctly identified re-infection in 36 of 52 patients (69%).

## Discussion

Determining the cause of recurrence in infectious diseases is important as relapse and re-infection have different implications for disease control and clinical management. Relapse reflects treatment failure, in which antimicrobial regimen, elimination of a persistent focus and drug adherence are the main concerns. This contrasts with re-infection, which involves exogenous infection with a new strain and therefore has implications for disease prevention and health education strategies. In clinical practice, cause and management of recurrent infection is highly complex and standard second-line drug regimen may be recommended where individualized retreatment schemes are not practical [16]. In recurrent melioidosis, if all recurrent episodes are assumed to be relapse due to failure of primary eradicative treatment (TMP-SMX based regimen), then inferior secondary treatment (amoxycillin-clavulanic acid) may be used despite the presence of an organism that is still sensitive to TMP-SMX [17],[18]. Use of inferior second-line drugs would unnecessary expose patients with re-infection to a higher risk of relapse from this new episode than would otherwise be the case [7]. In addition, non-medical treatment, the prevention of re-infection, remains ignored.

For many infectious diseases, the clinical differentiation of relapse from re-infection is difficult or impossible, and genotyping has generally been used for this purpose. Examples include tuberculosis [19],[20], malaria [21],[22], *Staphylococcus aureus* bacteremia [23], pneumococcal bacteremia [24], infective endocarditis [25] and nosocomial infections [26],[27]. Two typing methods were used in this study since MLST can resolve any uncertainty that arises during the interpretation of DNA macrorestriction patterns generated by PFGE [6]. The MLST scheme has been shown to confirm cluster assignments based on PFGE results in common organisms [28]–[30]. However, genotyping techniques are not widely available for tropical infections in endemic areas. In addition, isolates are rarely stored outside of the research setting, making it impossible to compare isolates associated with the primary and recurrent infection.

Clinical differences between re-infection and relapse have been proposed for Lyme disease, although a scoring system was not developed [31]. Scoring systems have been described to predict outcome from melioidosis [32], and to predict a number of other events including atrial fibrillation after cardiac surgery [33]. To our knowledge, our scoring system is the first clinically-based scoring system to differentiate between relapse and re-infection in any infectious disease. It is rapid and simple to use, necessitating data on only four easy to assess factors. This scoring index can be used where bacterial genotyping is unavailable, which covers nearly all melioidosis-endemic regions. The factors associated with recurrent melioidosis are similar to those reported for recurrence of Lyme disease (relapse after previous inadequate treatment and within a short period, and re-infection during the ‘high’ season when ticks increase in numbers) [31], and may represent features that could be used for assessing other infectious diseases.

Using genotyping to compare primary and recurrent isolates to distinguish between relapse or re-infection could be confounded by two major factors. First, ‘re-infection’ could actually represent relapse in the event that primary infection was caused by simultaneous infection with more than one bacterial strain, and different strains were picked by chance for genotyping [34]. This is unlikely in melioidosis since infection with more than one strain of *B. pseudomallei* occurs in less than 2% of cases [35]. ‘Re-infection’ could also actually represent relapse if genetic events occurred *in vivo* that led to alteration of one of the seven housekeeping genes that are sequenced in order to generate a sequence type. This would be predicted to be extremely unlikely as MLST is based on the sequence of housekeeping genes which are under neutral selection pressure [36]. Second, ‘relapse’ could actually represent re-infection in the event that re-infection was caused by a different strain that was nonetheless indistinguishable on genotyping from the first infecting strain. This would happen when infection sources were clonal or had limited genetic diversity, but this is highly unlikely in melioidosis as the *B. pseudomallei* population in the environment is extremely diverse [37].

Our finding of a non-linear association between duration of oral treatment received for the primary episode and predictive value of relapse is consistent with a previous analysis; patients treated for more than 20 weeks may have included those with a slow response to treatment or who had more complicated or severe disease associated with a higher risk of treatment failure and relapse [7]. Bacteremia and multifocal infection during the primary episode have been identified as risk factors for relapse compared to patients who did not have relapse [7]; however, these two variables were not significantly different between the relapse and re-infection groups. *B. pseudomallei* isolates obtain from patients with primary infection and re-infection were not resistant to amoxicillin-clavulanic acid and doxycycline, a finding that is consistent with previous studies [38],[39]. Acquired antimicrobial resistance in relapse organisms was also uncommon. A number of factors may relate to this: acquired resistant to ceftazidime is infrequent and related to fatal outcome during the acute episode of infection [40]; acquired resistance to carbapenems has never been observed in our patients; and patients who had incomplete treatment with oral eradicative drugs mainly abandoned their treatment due to drug side effects, which may not increase the risk of selection of resistance [1].

This scoring system will not affect prescribing practice relating to the initial treatment of recurrent melioidosis; standard first-line parenteral antimicrobials are recommended for the treatment of both relapse and re-infection as acquired resistance to either ceftazidime or carbapenems is uncommon. In general, first line oral eradicative treatment (TMP-SMX) should be used if the organism isolated is susceptible to this drug. However, the scoring system could help to identify the cause of recurrent melioidosis and may lead to individualized oral eradicative treatment and management. Patients with recurrent infection require a detailed history of initial treatment including duration of each drug used and compliance, and any lifestyle modification made by the patient that reduces exposure to environmental *B. pseudomallei*. For patients with predicted re-infection, first-line eradicative treatment should be used and education provided on prevention of further re-infection. For patients with predicted relapse, efforts should be focused on patient compliance and completion of a course of therapy of adequate duration. The second-line, less effective amoxycillin-clavulanic acid should be used in patients with relapse only where *in vivo* failure of TMP-SMX is considered possible.

We propose that this scoring system can provide timely and important bedside information where bacterial genotyping is unavailable, though it would be important to validate it in different settings, particularly those outside northeast Thailand.
